# Optimizing Botulinum Toxin Injections in the Platysma Muscle Based on Motor Nerve Distribution

**DOI:** 10.1111/jocd.70301

**Published:** 2025-06-26

**Authors:** Kyu‐Ho Yi, Isaac Kai Jie Wong, Jovian Wan

**Affiliations:** ^1^ Division in Anatomy and Developmental Biology, Department of Oral Biology, Human Identification Research Institute, BK21 FOUR Project Yonsei University College of Dentistry Seoul Korea; ^2^ You and I Clinic Seoul Korea; ^3^ The Artisan Clinic Private Limited Singapore Singapore; ^4^ Medical Research Inc. Wonju Korea

**Keywords:** aesthetic medicine, botulinum toxin, noninvasive

## Abstract

**Background:**

Platysmal bands contribute to neck ageing and lower‐face descent. Although botulinum toxin A (BoNT‐A) is approved for this indication, conventional whole‐muscle dosing requires many superficial injections with attendant risks.

**Objective:**

To develop an anatomically guided protocol that targets motor‐dense zones of the platysma, optimizing efficacy while reducing dose and complications.

**Methods:**

Sihler‐stained dissections mapped motor versus sensory innervation. Ultrasound‐guided injections were performed in 40 patients, delivering 30–60 U BoNT‐A per side at 15 sites concentrated in the upper two‐thirds of the platysma and along the marginal mandibular border. Outcomes included band severity, jawline definition, adverse events, and cost.

**Results:**

Motor end‐plates clustered in the upper platysma; the lower third was largely sensory. Targeted injections produced a ≥ 2‐grade reduction in dynamic bands in 92.5% of patients by week 4, maintained through week 12. Mean toxin use fell 35% versus historical whole‐muscle protocols; bruising dropped to 5%, with no dysphagia or lower‐face weakness. Treatment costs were proportionally lower.

**Conclusions:**

Concentrating BoNT‐A in the motor‐rich upper platysma under ultrasound guidance maximizes aesthetic improvements while minimizing dose, discomfort and adverse events. This neural distribution‐based algorithm offers a safer, more economical alternative to conventional platysmal band treatment.

## 1 Introduction

**FIGURE 1:**
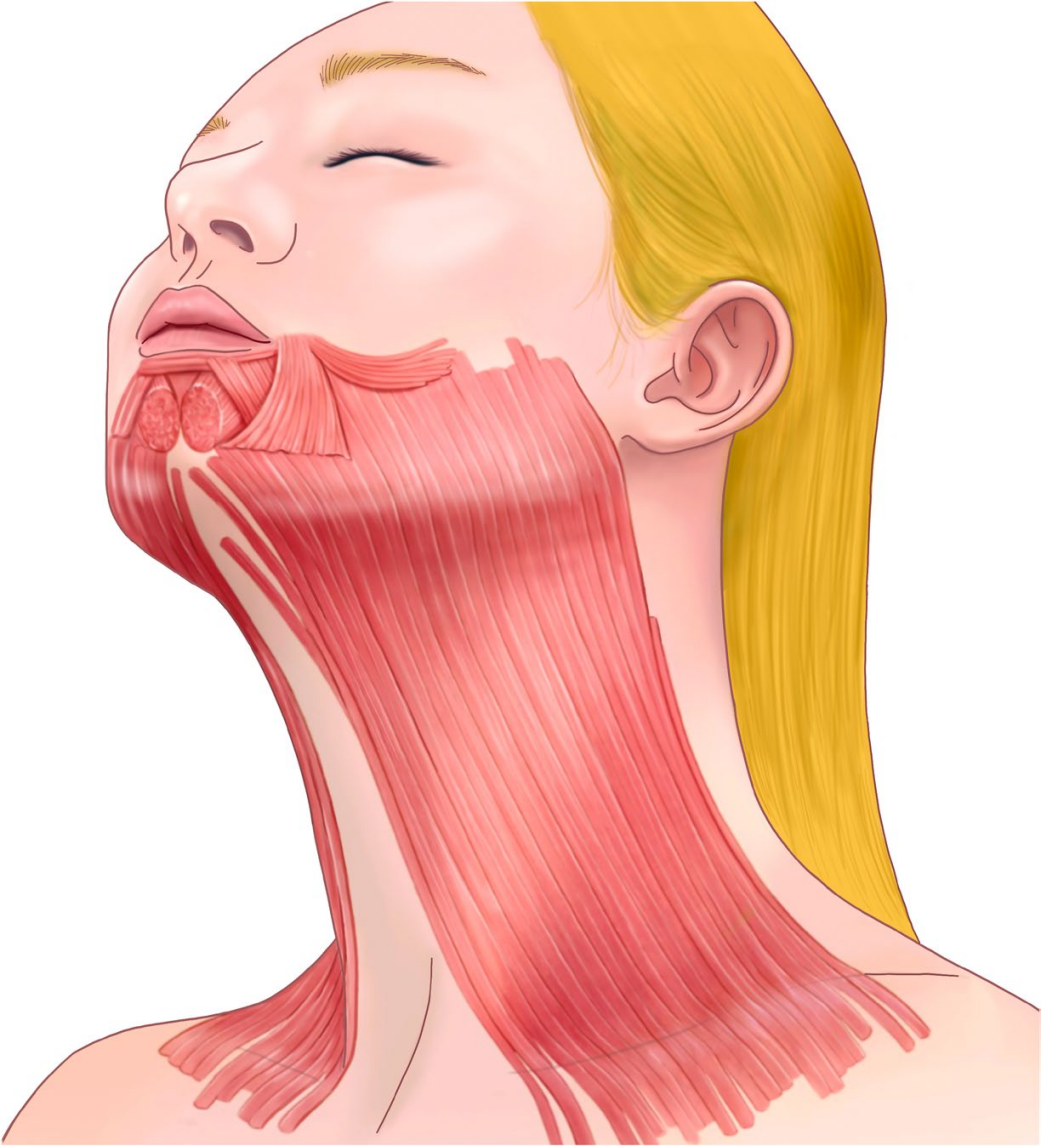
Schematic diagram of the platysma muscle.

The platysma muscle, situated in the anterior neck, plays a central role in the formation of dynamic vertical neckbands and acts as a depressor of the lower face. Its activity contributes not only to neck banding but also to downward traction on the jawline and lower facial contours, making it a key target in aesthetic rejuvenation procedures (Figure 1; Table 1) [1].

**TABLE 1:** Comparison of conventional versus neural‐guided botulinum toxin injection techniques for platysmal bands.

Aspect	Conventional technique	Proposed neural‐guided technique
Injection pattern	Multiple superficial intradermal/subdermal injections along the visible platysmal bands	Targeted injections at specific neural entry points of the platysma and marginal mandibular branches
Number of injection points	High (20–40 per side)	Low (15 per side)
Total toxin units required	Higher total dose (e.g., 60–100 units)	Lower total dose (e.g., 30–60 units)
Risk of bruising/pain	Higher due to multiple needle entries and superficial injections	Lower due to fewer injection points and deeper, targeted injections
Cost to patient	Higher due to increased toxin usage and more extensive procedure	Lower due to reduced toxin units and fewer injections
Immunogenicity risk	Potentially higher; superficial injections into dermis/subdermis may increase exposure to immune cells, raising the risk of neutralizing antibody formation	Potentially lower; deeper intramuscular injections may reduce exposure to immune surveillance, potentially decreasing the risk of antibody formation
Complication risk	Increased risk of dysphagia, dysphonia, neck weakness, and unintended diffusion to adjacent muscles	Reduced risk due to precise targeting of neural entry points, minimizing diffusion to non‐target muscles
Precision	Less precise; relies on surface anatomy and visual cues	More precise; guided by detailed anatomical knowledge of nerve distribution
Clinical validation	Widely practiced with established protocols	Emerging technique; requires further clinical studies for validation

Recently, botulinum toxin has received FDA approval for the treatment of moderate‐to‐severe vertical platysmal bands, establishing standardized dosing and injection patterns for this indication. While the approved techniques have demonstrated clinical efficacy, there remains potential for further refinement based on detailed anatomical understanding of neural innervation patterns. The neural‐guided injection approach proposed in this study is designed to complement existing standard practices by enhancing the precision of toxin delivery to key motor points of the platysma and marginal mandibular branches. Rather than replacing current recommendations, our method aims to improve treatment outcomes by optimizing muscle relaxation, reducing toxin dosage, minimizing complications, and contributing to both neck rejuvenation and facial lifting effects through anatomically informed injection strategies.

Conventional injection techniques for platysmal bands often require multiple superficial intradermal or subdermal injection points due to the wide, thin architecture of the platysma muscle. This increases the risk of bruising, patient discomfort, and post‐procedural pain. Furthermore, the need for a higher number of units to cover the extensive muscle surface area results in higher treatment costs for patients. From an immunological perspective, intradermal or subdermal injection into areas rich in immune cells may enhance the risk of neutralizing antibody formation against botulinum toxin, potentially diminishing treatment efficacy over time. This is because the dermis contains a greater density of antigen‐presenting cells compared to the deeper muscle tissue. Consequently, conventional techniques may inadvertently predispose patients to increased immunogenic responses, requiring more frequent treatments or higher doses in the future. Botulinum toxin type A (BoNT‐A) injections have long been used to relax the platysma, thereby reducing the appearance of these bands. Traditionally, injections have been administered across the entire muscle, but new anatomical insights reveal that focusing on the upper portion of the platysma, where motor innervation is concentrated, may achieve better results with fewer risks [2, 3, 4, 5, 6, 7, 8]. This clinical commentary explores the optimal injection points based on neural distribution and provides practical guidance for clinicians, supported by relevant figures.

### 1.1 Neural Distribution and Injection Strategy

The platysma is a superficial, broad muscle, extending from the lower face to the upper chest. It is primarily responsible for the formation of vertical bands on the neck due to muscle contraction. Anatomically, the platysma receives motor innervation from the cervical branch of the facial nerve, which supplies motor fibers predominantly to the upper half of the muscle. Additionally, the marginal mandibular branch of the facial nerve provides supplementary motor innervation to the uppermost part of the platysma near the jawline [9].

**FIGURE 2:**
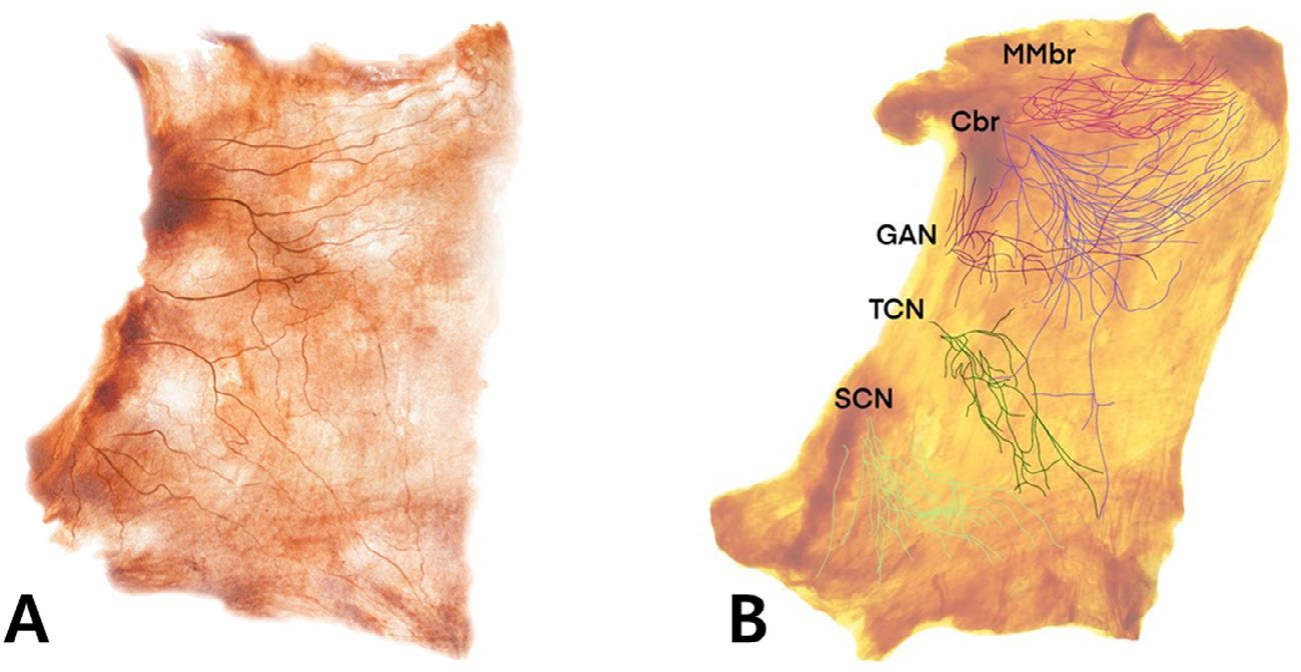
Shiler‐stained image of the platysma muscle revealing the neural distribution of the platysma muscle (A) Schematic diagram illustrating the neural distribution in the platysma muscle (B) The upper portion of the platysma is primarily innervated by the cervical branch of the facial nerve (Cbr) and the marginal mandibular branch of the facial nerve (MMbr), providing motor control to this region. The lower third of the platysma is innervated predominantly by sensory nerves, including the transverse cervical nerve (TCN), great auricular nerve (GAN), and supraclavicular nerve (SCN). These sensory nerves play little role in muscle contraction, making injections in the lower platysma less effective for treating dynamic neckbands.

The lower third of the platysma, by contrast, is innervated mostly by sensory nerves, including the transverse cervical nerve (TCN), great auricular nerve (GAN), and supraclavicular nerve (SCN). These sensory nerves play little role in muscle contraction, making injections in the lower platysma less effective for treating dynamic bands (Figure 2) [9].

### 1.2 Injection Points

Based on this neural distribution, BoNT‐A injections should be concentrated in the upper half of the platysma to effectively reduce muscle activity. Injecting BoNT‐A into the lower platysma, which is primarily innervated by sensory nerves, is unlikely to yield additional benefit and may increase the risk of complications.

### 1.3 Key Injection Points

**FIGURE 3:**
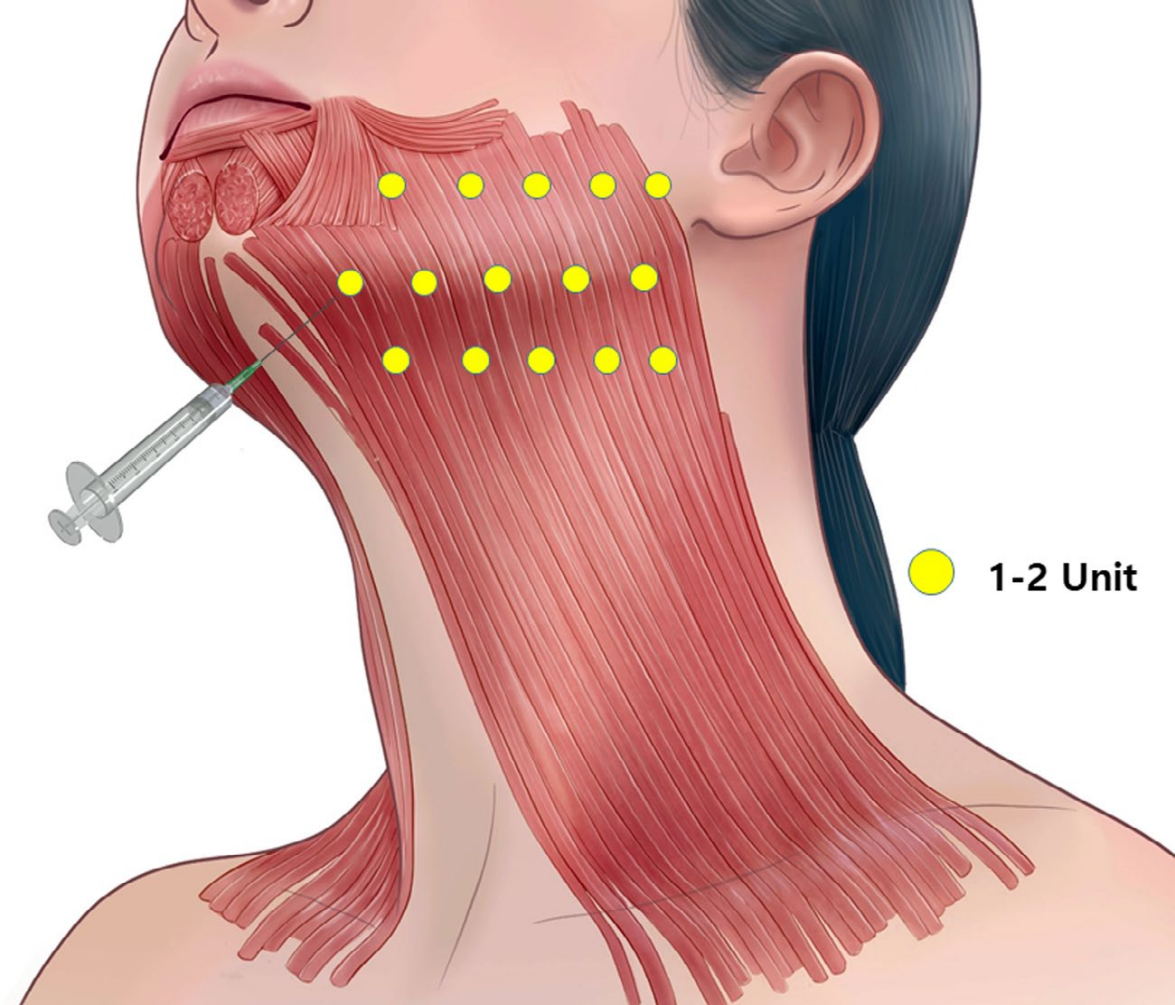
Anatomical distribution of injection points in the platysma muscle and marginal mandibular branch area. A total of 15 injection points per side are designated as follows: 5 points along the mandibular body, 5 points at the mandibular border, and 5 points approximately 1.5–2.0 cm below the mandibular border. BoNT‐A should be injected at 2–3 points along each vertical band in the upper half of the platysma muscle, indicated by green markers, to target motor innervation supplied by the cervical branch of the facial nerve. Additionally, small doses of BoNT‐A should be injected just below the mandible to address the uppermost neckbands in the marginal mandibular branch area. Superficial subdermal injections are used for the platysma fibers, while deeper injections target neural entry points at the mandibular border. Each injection site receives approximately 2–4 units of BoNT‐A (diluted at 2.5 units/0.1 mL), totaling 30–60 units per side. This structured approach aims to optimize treatment precision, minimize bruising and immune activation, reduce overall toxin dose, and achieve natural, long‐lasting aesthetic outcomes.

**FIGURE 4:**
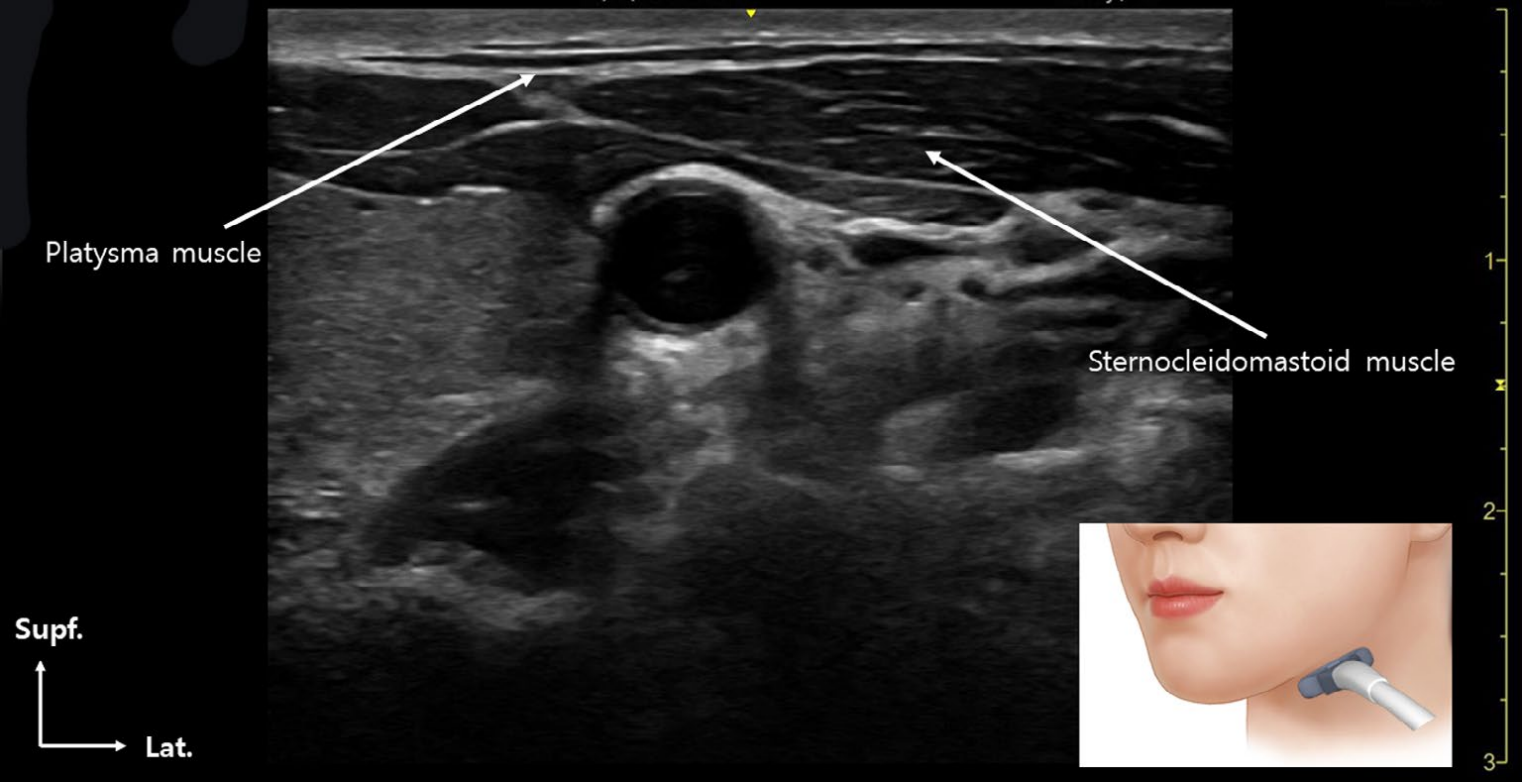
Platysma muscle observed under ultrasound guidance. This approach facilitates accurate botulinum toxin injection into the targeted muscle layer.

1. *Upper Platysma*: Injections should focus on the motor‐rich upper portion of the muscle. BoNT‐A should be injected along vertical bands formed by muscle contraction. Typically, injections are made at 2–3 points per band, spaced evenly across the upper two‐thirds of the neck. These points are usually located approximately 2–3 cm apart (Figure 3). *Marginal Mandibular and Cervical Branch Area*: In cases where banding extends closer to the jawline, additional injections may be necessary. The upper half region of the platysma, innervated by the marginal mandibular and cervical branch, often requires 1–2 injection points along the jawline to relax the platysma and define the neck‐to‐jaw transition (Figure 3).
2. *Avoiding the Lower Platysma*: Since the lower third of the platysma is largely innervated by sensory nerves, injecting BoNT‐A into this area provides minimal therapeutic benefit. Additionally, it poses a risk of weakening neck support muscles, leading to functional complications. Therefore, injections in this region should generally be avoided unless there is a clear anatomical indication.
3. Successful injection into the platysma muscle should ideally be performed under ultrasound guidance to ensure precise targeting along both the upper and lower jawline (Figure 4), enhancing accuracy and safety. In these regions, injections should be administered using a needle or cannula placed within the subdermal layer, rather than the intradermal plane, to access the muscle more effectively while minimizing surface trauma. While this technique does not aim for direct neural entry sites, it is anatomically guided by previously published Sihler's staining studies, which have mapped the neuromuscular junctions innervated by the cervical and marginal mandibular branches of the facial nerve. Given that distal nerve branches are microscopically small and not visualizable via ultrasound, the injection depth should be adjusted based on patient BMI, particularly in individuals with thicker preplatysmal fat. Although the therapeutic goal is intramuscular delivery, injecting slightly above the platysma in the subplatysmal or subdermal layer may offer a safer alternative, as injections below the muscle carry a greater risk of dysphagia or unintended paralysis of deeper cervical musculature.

## 2 Discussion

The anatomical insights regarding the neural distribution of the platysma suggest a more refined approach to BoNT‐A injections. Focusing on the upper half of the muscle, where motor innervation is most concentrated, offers the greatest potential for muscle relaxation and the reduction of vertical neckbands. By targeting the cervical branch of the facial nerve and, where necessary, the marginal mandibular branch, clinicians can achieve the desired aesthetic outcomes while minimizing the risk of side effects.

Avoiding injections in the lower platysma significantly reduces the risk of complications, including neck instability and excessive muscle weakening. Since the lower third of the platysma is predominantly innervated by sensory nerves, administering BoNT‐A in this region provides minimal benefit in terms of muscle relaxation [10]. Moreover, injecting into this area may compromise neck function, especially in older patients or those with underlying conditions that affect neck stability and strength. Therefore, focusing injections on the upper platysma, where motor innervation is concentrated, is a safer and more effective approach.

This targeted injection strategy also conserves the amount of BoNT‐A required, making the procedure more cost‐effective without sacrificing efficacy. By limiting injections to the upper platysma, clinicians can use smaller doses while still achieving significant improvements in neck appearance.

This study is based on clinical observations without a control group or randomized comparison to conventional injection techniques. Although the anatomical rationale and early clinical outcomes appear promising, the findings remain preliminary. Larger studies incorporating standardized outcome measures, objective functional assessments, long‐term follow‐up, and evaluation across diverse patient populations are necessary to validate the safety, efficacy, and cost‐effectiveness of the proposed neural‐guided injection protocol for platysmal band treatment.

## 3 Conclusion

In light of the motor innervation patterns of the platysma muscle, BoNT‐A injections should be concentrated in the upper half of the muscle, where motor control is densest. This approach minimizes the need for injections into the lower platysma, reducing the risk of complications and ensuring more efficient use of the toxin. The inclusion of targeted injection points and appropriate dosage distribution, as outlined in this commentary, provides a structured guide for clinicians aiming to achieve the best aesthetic outcomes while maintaining patient safety.

This refined injection technique is supported by anatomical evidence and is likely to become the preferred method for neck rejuvenation treatments using botulinum toxin. By focusing on the upper platysma and avoiding unnecessary injections into the lower portion of the muscle, clinicians can enhance both the safety and efficacy of the procedure.

## Author Contributions

All authors have reviewed and approved the article for submission. **Kyu‐Ho Yi, Jovian Wan:** conceptualization. **Kyu‐Ho Yi, Jovian Wan:** writing – Original Draft Preparation. **Kyu‐Ho Yi, Jovian Wan, Isaac Kai Jie Wong:** writing – Review and Editing. **Kyu‐Ho Yi, Jovian Wan:** visualization. **Kyu‐Ho Yi:** supervision.

## Consent

Informed consent was obtained from all participants, with full disclosure of the study's purpose, risks, and confidentiality.

## Conflicts of Interest

The authors declare no conflicts of interest.

## Data Availability

The authors have nothing to report.
